# From *Undang*-*undang Melaka* to federal constitution: the dynamics of multicultural Malaysia

**DOI:** 10.1186/s40064-016-3360-5

**Published:** 2016-09-29

**Authors:** Mohd Roslan Mohd Nor, Ahmad Termizi Abdullah, Abdul Karim Ali

**Affiliations:** 1 Department of Islamic History and Civilization, Academy of Islamic Studies, Universiti Malaya, 50603 Kuala Lumpur, Malaysia; 2 Termizi & Co, No. 29-2, Jalan 3/4C, Desa Melawati, Ulu Klang, 53100 Kuala Lumpur, Malaysia; 3 Department of Fiqh and Usul, Academy of Islamic Studies, Universiti Malaya, 50603 Kuala Lumpur, Malaysia

**Keywords:** *Undang*-*undang Melaka*, Multicultural society, Islamic history in Malaysia, Islamic ruling, Malaysian Federal Constitution, Peaceful co-existence

## Abstract

**Background:**

Malaysia is a multicultural state comprising three main races: Malays, Chinese and Indians. The three main religions are Islam, Buddhism and Hinduism. Other religions such as Sikhism and Christianity are also practised. Muslims are the majority comprising 67 % of the population.

**Methods:**

This paper is qualitative in nature. It applies historical comparative method in presenting its data. The *Undang*-*undang Melaka* (Malacca Laws) was obtained from the monograph available at National Library of Malaysia under the name of Hukum Kanun Melaka. Analysis was done on selected examples from this document.

**Result:**

This paper highlights that had there been no introduction to a common law system, Malaysia would have remained with its traditional laws influenced by Islam and its local customs as evident from *Undang*-*undang Melaka* (Malacca laws). The *Undang*-*undang Melaka* was practised from 1422 to 1444 and the law of the country was developed to accommodate the introduction of civil law during the colonial period.

**Discussion:**

One of the unique aspects of multicultural Malaysia is the fact that it has a parallel legal system: sharia and civil law. This paper examines histo-cultural development of the Islamic law as practised in pre-independent Malaysia, as well as the coexistence between these two laws after the independence of Malaya in 1957.

**Conclusions:**

This paper concludes that Islamic law in Malaysia is confined to Muslim family matters, while civil law covers all other matters.

## Background

Before the independence of Malaya (now Malaysia) in 1957, the country has its ruling system mainly based on religious guidance. The *sharia* or Islamic law has been incorporated in the existing states’ law, such as the *Undang*-*undang Kedah* (Kedah Laws), *Undang*-*undang Pahang* (Pahang Laws), and *Undang*-*undang Melaka* (Malacca Laws). *Undang*-*undang Melaka*, that has elements of *sharia*, was introduced during the reign of Sultan Muhamad Syah (1422–1444) (Fang 2007). One of the conditions of independence is the establishment of a legal system based on English common law, with Islam as the official religion of the Federation. After independence, Malaysia attempted to harmonise civil and religious laws in the government.

Islamic law, coupled with the various customary laws, is the foundation upon which the legal system came to be established (Shaik 1915). Islamic law, which at times is referred to as *hudud*, has been discussed in term of its implementation (Haneef 2010). One may agree with the argument that if colonisation had not been responsible for the introduction and application of English law in Malaysia, Islamic law would have become the law of the state (Bidin 2009; Ahmad 1999; Ibrahim and Yaacob 1997). However, the grant of the *Charters of Justice 1826* to the Straits Settlements, and the eventual application of English law both through the judicial process and through legislation in the Malay States had effectively displaced Islamic law from its premier position, to become limited (Ahmad and Rajasingham 2001).

Under the Federal Constitution of Malaysia, Islamic law is a matter over which the State Legislature has jurisdiction (Federal Constitution, Article 74, Ninth Schedule). Matters over which the State Legislatures have been permitted to make laws are:

Islamic law and personal and family law of persons professing the religion of Islam, including the Islamic law relating to succession, testate and intestate, betrothal, marriage, divorce, dower, maintenance, adoption, legitimacy, guardianship, gifts, partitions and non-charitable trusts; Wakafs and the definition and regulation of charitable and religious trusts, the appointment of trustees and the incorporation of persons in respect of Islamic religious and charitable endowments, institutions, trust, charities and charitable institutions operating wholly within the State; Malays customs; Zakat, Fitrah and Baitulmal or similar Islamic religious revenue; mosques or any Islamic public places of worship … (Temperman 2010; Hooker 2003).

With regards to Islamic criminal law, the Federal Constitution provides that the State Legislature may make laws for the:

… creation and punishment of offences by persons professing the religion of Islam against precepts of that religion, except in regard to matters included in the Federal List… (Federal Constitution, Article 74, Ninth Schedule).

Likewise, the State Legislature has jurisdiction over:

… the constitution, organisation and procedure of Syariah Courts which shall have jurisdiction only over persons professing the religion of Islam and in respect only of any of the matters included in this paragraph, but shall not have jurisdiction in respect of offences except in so far as conferred by federal law… (Federal Constitution, Article 74, Ninth Schedule).

Although it is true that the practices of Islamic law differed among the various Malay states due to the influences of custom (Abdullah et al. 2010), British intervention in the affairs of the Malay States had the effect of formalising the manner in which Islamic law was administered (Ahmad and Rajasingham 2001). Islamic law was left to be administered by the respective states, with the Sultans proclaimed as ‘Head’ of Islamic religion in each state, thus giving rise to the lack of uniformity in the administration of Islamic law in Malaysia, whereas the uniform application of English law throughout the land was guaranteed (Ahmad 1999).

The judicial power of the Federation resides in courts constituted under Article 121. Clause (1) of that Article mentions two High Courts of co-ordinate jurisdiction and status, that is, one in the states of Malaya known as the High Court in Malaya, and one in the states of Sabah and Sarawak known as the High Court in Sabah and Sarawak, together with lower courts as may be provided by federal law. Clause (1A) states that the court referred to in clause (1) shall have no jurisdiction in respect of any matter that falls within the jurisdiction of the Syariah Courts. The effect of Article 121 (1A) has been judicially considered in a number of cases whose decisions serve to further reinforce the fact that the role played by Islamic law within the system is limited, and is circumscribed by such powers and jurisdiction as may be conferred upon the Syariah Court and its officials under the various State enactments (Ahmad 1999). In a way, it confirms the establishment of two court systems, one related to Syariah matters and the other non-Syariah cases.

## Historical background of Islamic law in Malaysia

Historical background is significant in order to understand the basis of Islamic law in Malaysia. So far, the earliest record of Islamic law in Malaysia was found in an inscription on a stone in Terengganu called *Batu Bersurat Terengganu*, which dates back to 1303 (Al-Attas 1984; Nor et al. 2012; Halim et al. 2012). It mentions punishments for some offences following the provisions in the Qur’ān and *Sunnah*. For example, it is stated (in traditional Malay language):

Orang berbuat bala cara laki–laki perempuan titah Dewata Maha Raya jika merdehika bujang palu seratus rotan. Jika merdehika beristeri atau perempuan bersuami ditanam hinggakan pinggang dihambalang dengan batu matikan (Ibrahim 2002).

This is the law relating to the punishment for *zīnā* (adultery). It can be translated as:

Those who commit unlawful intercourse between male and female, the order by the God are: if they are free person (not a slave) and unmarried, they will be flogged a hundred lashes and if the free man has a wife or the free woman has a husband, they will be buried till waist and will be stoned to death.

This law is in accordance with traditional Islamic teaching. When Malacca was a Malay kingdom in early 15th century, a compilation of laws was made on the orders of the Ruler and this, the Malacca Laws (*Undang*-*undang Melaka*), shows the influence of Islam in Malay customary laws (Ibrahim 2000; Jusoh 1991).

The *Undang*-*undang Melaka* is a hybrid text (Fang 1976). In other words, it is composed of several separate texts bound together as one manuscript. It was copied and later recopied and although it undoubtedly came to be regarded as one text, the various component parts still clearly show themselves. The Malacca Laws consist of six different texts (Hooker 1986):

1. *The Undang*-*undang Melaka* (proper)
2. The Maritime Law (partly)
3. Muslim Marriage Law
4. Muslim Law of Sale and Procedure
5. *The Undang*-*undang Negeri*
6. *The Undang*-*undang Johor*

Based on the social traditions depicted by the *Sejarah Melayu* (Malay Annals), it is certain that Islam became the state religion of Malacca (Baharudin 2008). It is clear that in its administration, the rulers had appointed *Qādī*s (judges) as their advisers in religious matters. This was proven by the presence of prominent religious men such as Kadi Yusof, Kadi Monawar and Maulana Abu Bakar (Ismail 1998).

The Islamic laws in Malacca occupy almost a quarter of the sum of the local provisions, which concern marriage law, law of sale and procedure, and criminal law (Jusoh 1991).

Criminal law is mentioned in five chapters, Chapter 36, 39, 40, 41 and 42. The texts of these chapters are as below:

Chapter 36: Rules governing apostasy.

36.1 If a Muslim turns apostate, he will be ordered thrice to repent. If he refuses to repent, it is permissible, according to the law of God, that he be killed and (his body) should not be bathed and no prayer should be read (at his funeral) and (furthermore) he should not be buried in the Muslim graveyard.
36.2 Concerning people (who fail to perform) the (obligatory) prayer, there are two cases. (First), neglecting (the prayer) and (second), without firm belief in the obligatoriness of prayer. If a man (still) believes that prayer is obligatory, he is (simply) ordered to perform the prayer. If he does not perform the prayer due to illness, he has not turned apostate, but he will be ordered thrice to repent like an apostate. If he does perform the prayer, he will be pardoned. If he refuses to perform the prayer, he shall be killed but he is to be treated like a Muslim, and his body is to buried in the Muslim graveyard. Such is the law” (Fang 1976).

Chapter 39 Rules governing killing (*qiṣāṣ*).

39.1 If a person who is sane and of age wilfully kills a Muslim: whether the person killed is a man or a woman, whether (the person) is small or big, he who kills shall be killed.
  It is not permissible to kill a Muslim for killing an infidel: neither should a free man be killed for killing a slave; (nor) a father be killed for killing his son.
  If a Jew kills a Christian or even an infidel or a fire-worshipper (and the crime) remains unknown, (and) only later it becomes known, even if the Jew has become a Muslim, he is to be punished according to the law of God.

Chapter 40 Rules relating to unlawful intercourse (*zīnā*)

40.1 Concerning *zīnā* (unlawful intercourse) there are two cases: first, (that committed by) a man who is *muḥṣan* (legally married), i.e. a man who has been married in a legal marriage; (and second, that committed by) a man who is non-*muḥṣan* (non-married), i.e. an unmarried man or an unmarried woman.
  When *muḥṣan* he shall be sentenced to the *rajm* punishment and be stoned to death. When non-*muḥṣan*, the *ḥad* [*ḥudud*] punishment is that he shall be given one hundred lashes and expelled from the country for 1 year.
  Being *muḥṣan* means four things: first, a Muslim, second, of age, third, in full possession of his mental faculties and fourth, (he is) not insane.
  The *ḥad* punishment for a male slave or a female slave is half of that for a free man, that is, fifty lashes.
40.2 The punishment for sodomy and bestiality is the same as that for unlawful intercourse. If no actual intercourse has taken place, and the offence has gone no further than petting, the judge will pronounce a *ta‘zīr* punishment for the offender, and (if) the judge gives the *ḥad* punishment, it is twenty lashes only.
  Unlawful intercourse is punished by the judge, if (the offender) has made a confession or when four male witnesses (who are) free men have caught the offenders in *flagrante delicto* (while the crime is flagrant, in the very act of committing an offence).
  If two witnesses say, “We saw him committing unlawful intercourse on one corner” (and) two other witnesses declare, “We saw him committing unlawful intercourse in another corner”, (the offence) is not proven. With regard to the rules governing unlawful intercourse, the four witnesses must agree with one another, only then is the unlawful intercourse proven, (and the offender) shall be given the usual punishment mentioned above.

Chapter 41 Rules relating to slander.

41. (If) a man slanders another man and the slanderer denies because there were no witnesses present, the slanderer shall be scourged eighty lashes.
  If the slanderer is a slave, he shall be given forty lashes. When (the slander) occurs between a slave and another slave or an infidel, the slanderer shall be imprisoned until he is given a *ta‘zīr* punishment by the judge.

Chapter 42 Rules relating to alcoholic drinks.

42. (Concerning) anyone who drinks alcoholic drinks or any drink which is intoxicating: if a free man, he shall be scourged forty strokes; if a slave twenty strokes.
  The *ḥad* punishment is given on the basis of two things; first, a confession (by the offender); (and second), when there are two male witnesses. The *ḥad* punishment shall not be inflicted, if someone’s mouth just smells of alcohol, that is, he (the man) shall not be sentenced (Fang 1976).

Hence, it can be seen that a dual system of law (customary and Islamic law) was practised. Therefore, it is noted that in many chapters of the law relating to punishment for a certain crime, after prescribing the penalty according to *hukum adat* (the law of local custom), the text also mentions various alternative penalties according to ‘*hukum Allah’* (the law of God) (Jusoh 1991).

For instance, it is mentioned in Chapter 5.3: relating to the killing of a paramour, it is worth to highlight the reference from the *Undang*-*undang Melaka*. It says (in traditional Malay language):

Adapun jikalau membunuh madu, maka madunya itu lari ke dalam kampung orang, tiba–tiba maka diikutnya jua oleh empunya madu itu, maka berkelahi dengan orang yang empunya kampung itu, maka melawan ia, maka terbunuh oleh orang yang empunya kampung itu, mati sahaja tiada dengan hukum lagi. Itulah adat hukum dalam negeri, tetapi kepada hukum Allah, tiap–tiap membunuh itu dibunuh juga hukumnya, kerana menurut dalil di dalam Qur’ān dan menurut amru bil-ma‘rūf wan-nahyu ‘anil-munkar (MSS23 Hukum Kanun Melaka (n.d.)).

It is translated as:

If he (a paramour) runs into someone’s compound and is pursued by the husband, whereby the latter is involved in a fight with the owner of the compound; If he (the owner of the compound) resists him and the pursuer is killed, the latter simply dies and there shall be no litigation. This is the custom of the country. But according to the law of God, he who kills shall also be killed. For this is in accordance with what is stated in the Qur’ān and is in pursuance of (its teaching): (God bids us) to do good, and forbids us to commit sin (Fang 1976).

A similar feature of the law also appears in chapter 7.2 regarding theft, chapter 8.2, 8.3 and 8.4 pertaining to slapping and killing respectively. The law on killing is also described in chapter 18.4 while The law relating to fornication (*zīnā*) is described in chapter 12.2. The “law of God” is prescribed instead of the *Kanun* Law (a term referring to *Undang*-*undang Melaka*) for the crime of killing (chapter 5.1) and theft committed in a house (chapter 11.1). However, the Law of God as mentioned in chapter 10, 11.4, 14.2 and 16.1 contradict Islamic teachings (Jusoh 1991). Chapter 14.2 (concerning accusation of seizing another’s wife) is an example. It is stated:

If the accused succeeds (in pleading innocent), the accuser shall be sentenced to death, because the punishment for seizing another’s wife is death. If he (the accuser) is not sentenced to death, he shall be fined 10 ¼ tahil. Meanwhile only the judge can intervene (to save the accuser), because the punishment (for this offence) is death. But according to the law of God, it is not so, he (the accuser) is ordered by the judge only to swear the oath and repent his deed. Such is the law of God (Fang 1976).

According to Hamid Jusoh (1991), this religious terminology may have been used as a psychological tool to mislead society. He wrote that certain parts of the first 24 chapters are in conflict with Islamic criminal law and procedures described in chapters 36-42. The differences in these provisions maybe due to the different time period when the respective laws were written and compiled. It might have also been due to different attitudes of society towards the acceptance of Islamic law (Jusoh 1991).

According to Fang (1976), it is futile to attribute the compilation of *Undang*-*undang Melaka* (Malacca Laws) to any one ruler or at any one period. Nevertheless, the Malacca Laws (proper), and possibly also the section on Maritime Law, were first established during the time of Sultan Muhammad Shah (1424-44) and were completed during the reign of Sultan Muzaffar Shah (1445-58), the golden period of the Malacca Sultanate. The section on Muslim (Islamic) laws, especially those pertaining to commercial matters and procedures, (including criminal) may have been compiled sometime later (Fang 1976).

Again, according to Hamid Jusoh (1990), Malacca Laws failed to outline the rules and the country’s administration clearly. It only stated some of the Islamic principles relating to the rulers and the concept of leadership. On the other hand, Mahmood Zuhdi commented that, even if the matters relating to the Federation were not mentioned in Malacca Laws, this does not mean that absenteeism of the said laws in writing can be taken as grounds to say that the basis of Islamic constitution never existed at that particular time. This is because the constitution for a federation may not necessarily be in writing. It may have been an oral tradition (Mahmood Zuhdi 1997).

When the kingdom of Malacca was defeated by the Portuguese in 1511, the texts of the Malay laws were taken and adapted with modifications in the various Malay states including Pahang, Johore and Kedah (Ibrahim, 2000).

The Pahang laws which were formulated during the reign of Sultan Abd. Ghafur Muhaiyyuddin Shah (1592–1614 A.D.) following the *Undang*-*undang Melaka*, the present authors found that the influence of the Malay custom was less than the Islamic law which was generally followed. Thus, there were provisions based on the Islamic law dealing with *qiṣāṣ* (section or s. 46 and 47), fines (s. 48), unlawful intercourse (s. 49), sodomy (s. 50), theft (s. 53), robbery (s. 54), apostasy (s. 62), omission to pray (s. 60), jihad (s. 61), procedure (s. 62), and witnesses and oaths (s. 64). There were also provisions dealing with trade sale, security, guarantee, investments trust, payment for labour, land, gifts and *waqf*s.

The Johore laws too, were modelled on the *Undang*-*undang Melaka*. At the beginning of the 20th century, the codifications of the Islamic law, as seen in Turkey and Egypt, were translated into Malay and adopted. The *Majallah al*- *Aḥkām* was as adapted as the *Majallah Aḥkām Johor* (Borham 2002) and the *Ḥanafī* code of *Qadri Pasha* was adapted and translated as the *Aḥkām Shar‘iyyah Johor* (Ibrahim and Joned 1995).

However, Mahmood Zuhdi (1997) commented that even *Majallah al*-*Aḥkām* was copied from Turkey; it was introduced during a period where Johore’s legal system was heavily influenced by the British. It can also be argued if *Majallah al*-*Aḥkām* was a true reflection of the Islamic law or if had been widely influenced by the legal system in Europe at that particular time (Mahmood Zuhdi 1997).

According to Ahmad Ibrahim, all these examples show that there were attempts before the arrival of the British to modify the Malay customary laws and to adopt Islamic laws. This was in progress when the British arrived and exercised their influence in the Malay states (Ibrahim and Joned 1995).

In the case of *Shaikh Abd. Latif* v. *Shaikh Alias Bux* [(1915) 1 FMSLR 204], the court held that the only law applicable to Malays was Mohammedan law modified by local customs. However, according to Hooker (1984), in this instance, Muslim law was applied, not because it was the law of Selangor but because it was the proper law relating to a Muslim estate. It was ‘the personal religious law of the Muhammadan inhabitants …’ and it was applicable in the Courts of the Federated Malay States because the Crown had bound itself by treaty not to interfere with issues related to Malay customs and the Muslim religion. In other words, Hooker was saying the Court was not prepared to admit that Muslim law had a territorial definition as the law of a State or an area.

In this case, the present authors are incline to support his view by mentioning the case of *Ong Cheng Neo* v. *Yap Kwan Seng* [(1897) 1 S.S.L.R. Supp. 1]. In this case it was held (a) that ‘English law as such does not prevail in these Courts except insofar as it has been adopted’, and (b) ‘… the entire Muhamedan law is a personal law … it gives rights only to those who acknowledge Islamism. Only a Muslim has a right of succession regulated by the laws of Islam’ (Hooker 1984).

In the case of *Ramah binte Ta‘at* v. *Laton binte Malim Sutan,* [(1927) 6 F.M.S.L.R. 128 (CA)] the majority of the Court of Appeal held that the Muslim law was not foreign law but local law and the law of the land of which the court must take judicial notice. The court must accept the law and there should be no confusion as to what was the local law. However, M. B. Hooker in commenting this case wrote:

However, as the judgment makes clear, the Court really had no idea of what the rules of this so-called ‘local law’ were in respect of the issue before it. It was, in other words, placed in the embarrassing position of attempting to administer a local law without knowing its contents! (Hooker 1984).

## Replacing Islamic Law

The British arrival in Malaya led to the introduction of the English law thus replacing the hitherto Islamic law. Malaya was officially colonised by the British in 1824 via the Anglo-Dutch Treaty of 1824, which divided the Malay Archipelago into British and Dutch spheres of influence. Malacca and the rest of the Malay states came under the jurisdiction and administration of the British while Indonesia was governed by the Dutch.

Penang and Malacca became British colonies and therefore the English law was introduced via the *Charter of Justice 1826*, which set up the courts of judicature and provided in effect that the Courts should apply the English law. Nonetheless, this was subject to the qualification that in cases where the introduction of English law would cause hardship and injustice to the inhabitants, they would be allowed to follow their own personal law. Thus, in regard to the Muslims in Malacca and Penang, they were allowed to follow the Muslim law in matters of marriage and divorce. Later, legislation was enacted to provide for the administration of the Muslim family law.

The Malay States were, in theory, independent Malay kingdoms but English law was introduced through the influence of the British in two ways: first, under the treaties made by the Malay Sultans with the British, the Sultans agreed to receive British Residents or Advisers in the states and to follow their advice in all matters of administration except in relation to the Muslim religion and Malay customs. Under these provisions, the British Resident advised the Sultan to enact laws such as the *Contracts Act*, the *Penal Code*, the *Evidence Act*, the *Criminal Procedure Code* and the *Civil Procedure Code*, based on the Indian modifications of English law and in the case of land, laws following the legislation in Australia based on the Torrens system of registration of title.

The second stage of British influence was the introduction of the court system. On the advice of the British, the Malay Sultans set up civil courts and these were presided by British judges. In the absence of legislation governing this matter, the judges tended to refer to the law in England and in this way the English law of torts and the English rules of equity were introduced to the Malay States. The end result was the English law replaced the Islamic law in many matters and this was confirmed by the civil law legislations, culminating in the *Civil Law Act 1956*, provided in section 3 and 5 (Ibrahim 2000).

British colonialism and its influence led to Peninsular Malaysia inheriting a dual system of courts. The civil courts deal with the majority of the laws concerning contracts, torts, commercial cases, property and succession to property, crime and constitutional and administrative cases; all Malaysians are subjected to the jurisdiction of the civil courts. The Syariah Courts on the other hand deal mainly with Islamic family law and some criminal offences relating to the practice of Islam, and have jurisdiction only over Muslims. An amendment to the Federal Constitution has provided that the civil High Court and subordinate courts shall have no jurisdiction over any matter that falls within the jurisdiction of the Syariah Court (Federal Constitution, Article 121 (1A)). The jurisdiction of the Syariah Courts however, is still limited (Ibrahim 2000; Faruqi 2005; Mohamad 2008; Salbiah 2005). Hence, some would see these two separate legal systems as not harmonious and thus, more serious efforts need to be taken to harmonise them (Pa et al. 2010).

## Islam under the Malaysia’s Federal Constitution

A country’s Constitution plays an important role in its administration. However, most of the time the Constitution does not spell out everything in detail. For example, Malaysia’s Federal Constitution has not mentioned ‘the powers of the government’ or ‘the powers of the Prime Minister’… Thus, what is known as the unwritten constitution, namely the practice, convention, or the tradition of the constitution have been the fact.

Due to this uncertainty, the role of the court becomes important. This becomes apparent during the litigation process where the courts determine and validate (or invalidate) certain practices. Nevertheless, it is not rare that the judgements by the court are more confusing and fail to solve the problem.

The same scenario prevails regarding the position of Islam in the Constitution; a matter that has to be discussed first before discussing the issue of Islamic criminal law. The position of Islam in the Constitution is generally limited. Article 3 (1) of the Federal Constitution recognises Islam as the religion of the Federation. It says:

Islam is the religion of the Federation; but other religion may be practised in peace and harmony in any part of the Federation.

Though Article 3 (1) recognises Islam as the religion of the Federation, subsection 4 of the same Article limits it. It says:

Nothing in this Article derogates from any other provision of this Constitution.

Malaysia has two legal systems—civil and Syariah—and the Syariah Court has limited jurisdiction, as seen in in the above sub section. Nevertheless, the status of Islam compared with other religions—which are free to be practised by their followers—is at a higher level and recognised by the government (Bari 2002).

Article 12 (2) of the Federal Constitution gives rights “for the Federation or a State to establish or maintain or assist in establishing or maintaining Islamic institutions or provide or assist in providing instruction in the religion of Islam and incur such expenditure as may be necessary for the purpose” (Federal Constitution, Article 12 (2)). In the case of *Meor Atiqulrahman bin Ishak & Ors* v. *Fatimah Bte Sihi & Ors,* (MLJ, 2000, 5: 375) it was interpreted that ‘Islam is the religion of the Federation, but other religions may be practised in peace and harmony…’ as Islam is the principal religion compared with other religions which are practised in this country such as Christianity, Buddhism, Hinduism and others. Thus, Islam in Malaysia occupies a superior position. It may be because of this position, it appears that some minority groups and open-minded Muslims feel increasingly discriminated despite the apparent protection provided by the Federal Constitution (Jeyamohan 2004; Haji 2014). However, whether Islam, the official religion, overshadows the civil courts remains to be seen.

According to Hairuddin Megat Latif, the position of Islam in Malaysia’s federalism is not very important. This is based on the fact that the said powers are mentioned under the State List of Ninth Schedule of the Federal Constitution, while important matters such defence, internal security, criminal law and the administration of justice and finance are listed under the Federal List (List I—Federal list (Legislative Lists) of Ninth Schedule of the Federal Constitution) (Latif 1992).

Based on this provision, the power to legislate any laws relating to Muslims regarding the matters included in the State List is within the jurisdiction of the state. Thus, every state has the power, and because of this, it is a fact that the Islamic Law Enactments are different from one State to another. Moreover, the *fatwa* relating to some issues are different between the States. These different Islamic Law Enactments are not only confusing the public but also affecting the firmness of the *fatwa* itself and the Muslim image as a whole.

The effect of the lack of uniformity of these laws can be seen from the different time when Hari Raya Aidilfitri is celebrated. This occurred in 1982, when the state of Perak celebrated it one day earlier from the date announced by the King (*Yang di*-*Pertuan Agong*—*YDPA*). The difference can also be seen from the *madhhab* (school of thought) point of view when for example, the state of Perlis does not state the madhhab it follows while other states follow the *madhhab* of Shāfi‘ī (Latif 1992).

Although Islam is the religion of the Federation, there is no head of Muslim for the entire Federation. The King (*Yang di*-*Pertuan Agong*—*YDPA*) is the Head of Islam in his own state and in the Federal Territory, Malacca, Penang, Sabah and Sarawak as these states have no Malay Rulers of their own. The King’s representatives in these states, known as *Yang di*-*Pertuan Negeri*, are effectively the patrons of Islam. The remaining nine states of Malaysia have each their own Ruler, or Sultan, as the Head of Islam in that states. The Conference of Rulers have agreed, however, that in respect of ceremonies and observances in the Federation, the King is authorised to represent each of the Rulers as the head of religion in their states [Federal Constitution, Article 3 (2)].

The various state constitutions provide that the Ruler may act in his discretion in the performance of any functions as the patron of Islam, but it appears that the *YDPA* may only act on advice in performing his functions as the patron of religion in Malacca, Penang, the Federal Territory, Sabah, and Sarawak (Kamali 2000).

Though the state government has the power to enact laws for Muslims it only relates to and governs personal laws and family matters. Criminal matters also, depend on the *Syariah Courts (Criminal Jurisdiction) Act 1965* (revised 1988 (Act 355)). This means that even if the state governments can enact laws to govern Islamic criminal matters, their jurisdictions are still limited. For example, a Muslim who commits *zinā* cannot be punished as prescribed by the Qur’ān (24:2).

By virtue of the said Act, the punishments that can be imposed by the Syariah Court for criminal offences (including *zinā*) under Sect. 2, *Syariah Court (Criminal Jurisdiction) Act 1965*, are as below:

1. imprisonment not more than three years, or
2. fine not more than RM 5000, or
3. whipping not more than six strokes, or
4. the combination of all those punishments

The state government is neither able to implement or enforce the Islamic law other than what has been mentioned under the State List because it is against Article 74 (2) of the Federal Constitution, which reads as:

Without prejudice to any power to make laws conferred on it by any other Article, the Legislature of a state may make laws with respect to any of the matters enumerated in the State List (that is to say, the Second List set out in the Ninth Schedule) or the Concurrent List.

If this happens, the said laws will be regarded as invalid by the court because the laws, which are against the Federal Constitution, are void. This is mentioned in Article 4 (1) of the Federal Constitution, which reads as:

This Constitution is the supreme law of the Federation and any law passed after Merdeka Day which is inconsistent with this Constitution shall, to the extent of the inconsistency, be void.

Thus it is clear that Islamic laws are only accepted to be implemented in a very limited way, and even this limitation is subject to intervention (Jusoh 1990). Hairuddin Megat Latif concluded that, this situation is not so strange because the main objective of making Islam as the official religion of the Federation is only for formal functions such as to allow prayers to be performed in a good manner, and the coronation function of the *Yang di*-*Pertuan Agong*, among others (Latif 1992).

## Conclusion

Based on the above discussion, it can be discerned that historically, the laws which were implemented in Malaysia before colonisation, were mainly based on Islamic influence. Some of them were in harmony with Islam even though they were not based on the Quran or Hadith (they were based on customary laws). If we were to compare the position of Islam during the Malacca Sultanate and its current position, the scope in the Malacca Laws was wider as the laws were not only limited to the personal and family matters but also covered aspects of crime, civil, economy and commercial. The changes and developments to the Muslim laws were also influenced by the pluralistic society as well as respect for multiculturalism in Malaysia.
